# Dr. Thomas D. Strong

**Published:** 1911-07

**Authors:** 


DR. THOMAS D. STRONG, one of the best known physicians in Chautauqua county, died at his home in Westfield on June 5, 1911, at the age of 89 years. Dr. Strong was graduated from the University of Buffalo, medical department, in 1851, and spent the greater part of his life in Western New York.

